# UV Radiation and Skin Cancer: The Science behind Age Restrictions for Tanning Beds

**DOI:** 10.1289/ehp.120-a308

**Published:** 2012-08-01

**Authors:** Charles W. Schmidt

**Affiliations:** **Charles W. Schmidt**, MS, an award-winning science writer from Portland, ME, has written for *Discover Magazine*, *Science*, and *Nature Medicine*.


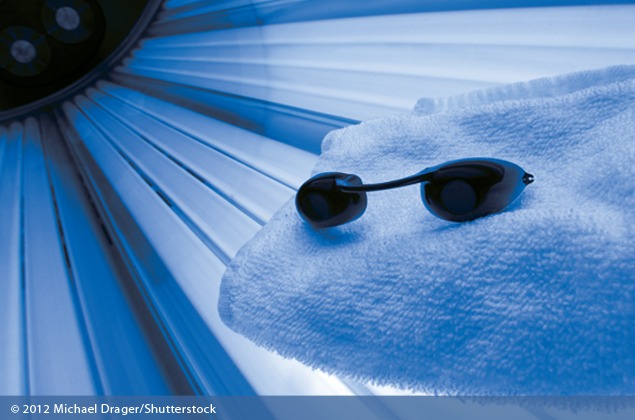
Every year, millions of people climb in various states of undress into warm, glowing tanning beds, where during a typical 2- to 15-minute session they’ll absorb a controlled dose of ultraviolet (UV) radiation at an intensity up to two to three times stronger than the sunlight striking the equator at noon. The tanning industry has grown rapidly since the 1980s,^^1^^ rising to an estimated 28 million users in the United States.^^2^^ This rise has been accompanied by an increase in diagnoses of skin cancer.

The reasons behind the rising skin cancer diagnoses remain open to debate. Some experts attribute the rise to more frequent skin cancer screening, whereas others blame environmental and behavioral risk factors, particularly changes in UV exposure. In this latter context, UV-emitting tanning beds—classified as carcinogenic to humans by the International Agency for Research on Cancer (IARC)^^3^^—have come under growing scrutiny.

People tan to look healthy, but looks can be deceiving; UV radiation causes all three types of skin cancer. Melanoma, a tumor of the cells that produce the skin pigment melanin, is the rarest but deadliest type, accounting for 75% of skin cancer deaths worldwide.^^4^^ According to the National Cancer Institute’s Surveillance, Epidemiology and End Results (SEER) program, melanoma incidence among U.S. whites (who develop the disease more often than other races) rose from 8.7 cases per 100,000 people in 1975 to 28 cases per 100,000 in 2009.^^5^^ Most of that increase occurred in older men, who rarely tan indoors. But a closer look at the age-stratified SEER data reveals that melanoma rates among white girls and women aged 15–39 rose by 3.6% per year between 1992 and 2006, compared with a 2% increase per year among boys and men of the same ages.^^6^^

Although they’re not tracked by SEER, squamous cell carcinoma (SCC) and basal cell carcinoma (BCC)—the other two types of skin cancer—also appear to be on the rise, according to regional studies from the United States and Europe. A recent study by Anne Marie Skellett, a consulting dermatologist at Norfolk and Norwich University Hospital, reveals that BCC diagnoses among people under age 30 in the United Kingdom jumped 145% between 1981 and 2006.^^7^^

Statistics such as these have prompted 33 U.S. states and some municipalities to ban or restrict indoor tanning among children under age 18.^^8^^ California’s ban, signed into law in October 2011, was the first,^^9^^ followed by Vermont in April 2012^^10^^ and the city of Chicago the following June.^^11^^ Other states have introduced legislation to limit indoor tanning among minors.^^8^^

**Figure:**
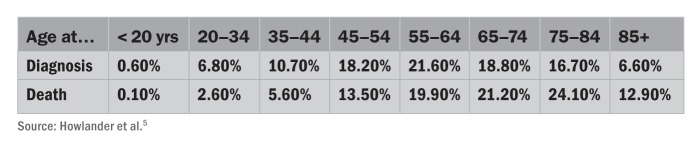
**Melanoma in the United States** From 2005 to 2009, the median age at diagnosis for melanoma of the skin was 61, and the median age at death was 68. The age-adjusted incidence rate was 21.0 per 100,000 men and women per year. Based on melanoma rates reported from 2007 to 2009, 1.99% of men and women born today will be diagnosed with melanoma of the skin at some point in their life.

**Figure:**
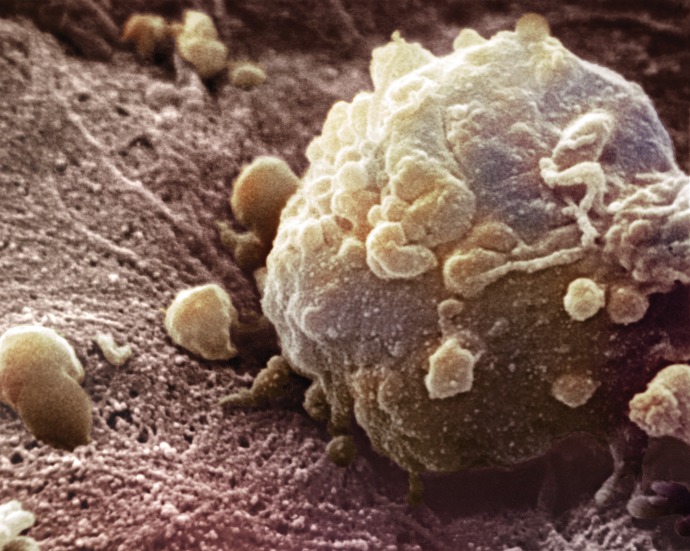
Scanning electron micrograph of a melanoma cell magnified 8,000 times © 2012 Biophoto Associates/Photo Researchers, Inc

Mary Brady, an associate professor of surgery at Weill Medical College in New York and the author of an editorial on indoor tanning that appeared in the May 2012 issue of the *Journal of Clinical Oncology*,^^12^^ says the bans make sense. “We legislate against smoking in kids less than 18, and that sends a strong message that there’s something wrong with it,” she says. “We need to send the same message on indoor tanning.”

But the bans have drawn a backlash from the tanning bed industry, whose representatives say they’ve been unfairly and incorrectly singled out. John Overstreet, executive director at the Indoor Tanning Association in Washington, DC, describes the evidence linking indoor tanning to skin cancer as speculation and advocacy science reported by the media as fact. He points out that UV light triggers skin cells to produce vitamin D, which may have cancer-protective effects. “It’s frustrating,” he says. “There’s no doubt that repeated overexposure to UV or burning can cause skin problems, but you also have to look at the health benefits, and that issue always gets lost.”

## Assessing the Weight of the Evidence

Artificial UV radiation made its public debut in the 1940s, used for promoting vitamin D synthesis in children. Early devices were heavily weighted toward shorter-wave UVB radiation, which produces vitamin D but can easily burn skin. By the time indoor tanning became popular in the 1980s, the trend was toward longer-wave UVA rays that don’t burn skin so readily.^^13^^ IARC describes UVB as a “complete human carcinogen” because of its ability to cause direct DNA damage. UVA, on the other hand, is carcinogenic by an indirect mechanism: It’s involved in the production of DNA-damaging free radicals, such as hydrogen peroxide.^^13^^ Overstreet says most of the tanning beds used now in the United States emit a ratio of 95% UVA to 5% UVB.

Scientists started investigating potential links between artificial UV exposure and skin cancer during the late 1970s. IARC reviewed 19 such studies during a meta-analysis published in 2006.^^13^^ The results showed a 15% increased risk for melanoma, 125% increased risk for SCC, and 3% increased risk for BCC among those who had ever tanned indoors compared with those who had never done so. In a subset analysis, IARC looked at seven studies that homed in on melanoma in relation to age at first incidence of indoor tanning. That separate analysis revealed a 75% higher risk among people who had ever tanned indoors before age 35 compared with those who never tanned indoors.^^13^^ It was on that basis, combined with sufficient evidence of an increased risk of ocular melanoma associated with the use of tanning devices, that IARC classified indoor tanning as carcinogenic to humans in 2009.^^3^^

Kelly Stoddard, state vice president of health and advocacy initiatives with the American Cancer Society in Williston, Vermont, says IARC’s widely publicized move to classify indoor tanning as carcinogenic is in part what motivated her to spearhead the Vermont ban. “We have data showing that 21% of young women in Vermont use tanning beds,” Stoddard says. “And melanoma rates in the 25- to 29-year age group here are growing, which leads us to think it has something to do with UV damage during the teen years.”^^14^^

**Figure:**
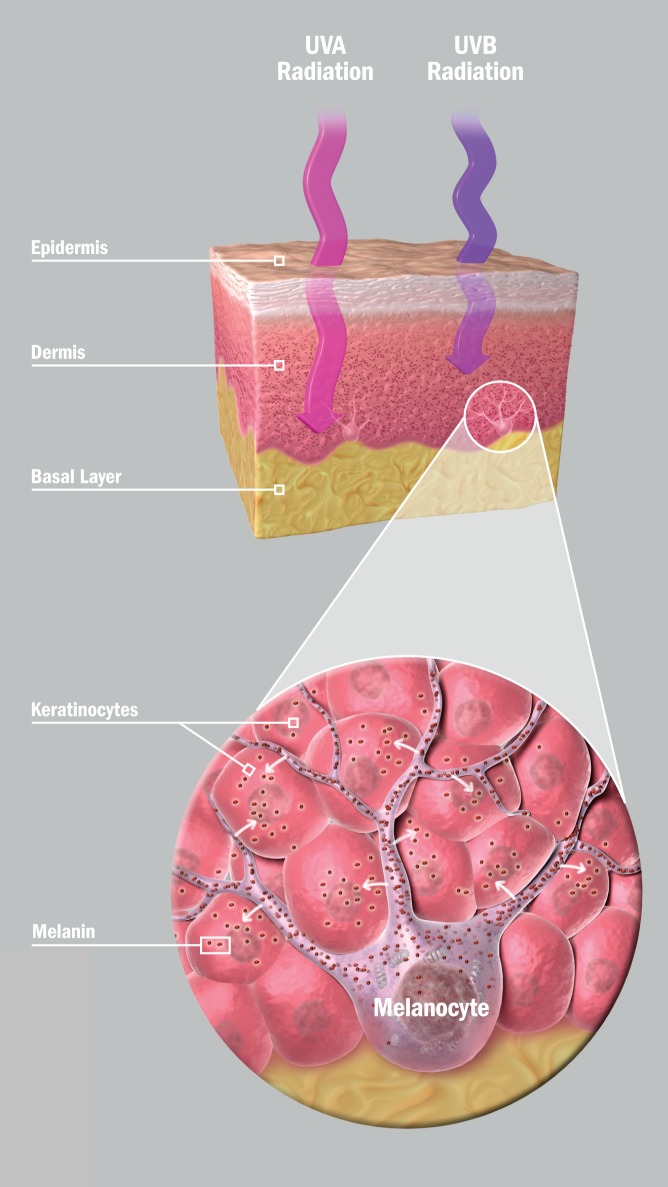
Melanocytes are skin cells that produce melanin, the pigment that gives skin its color. Short, intense waves of UVB radiation stimulate organelles known as melanosomes to produce melanin, which is absorbed by surrounding keratinocytes. Longer waves of UVA radiation penetrate more deeply into the skin and reacts with melanin, turning it brown. © 2012 Gary Carlson

But although the IARC classification was influential in terms of state health policy, the analysis on which it was founded also suffered from what many experts say is a significant shortcoming: inadequate exposure assessment. According to DeAnn Lazovich, an associate professor at the University of Minnesota School of Public Health in Minneapolis, none of the studies included in the analysis measured dose response the same way, and most of them didn’t account adequately for the confounding effects of sun exposure. What’s more, the studies were limited by the fact that early tanning devices emitted UVB radiation at levels much higher than those in use today.

More recent studies have sought to address those shortcomings, including one published by Lazovich and colleagues in 2010. For their analysis, Lazovich’s research team gave questionnaires to 1,167 patients diagnosed with invasive cutaneous melanoma in Minnesota between 2004 and 2007, and to 1,101 matched controls, in order to assess their indoor tanning habits. The team collected a broad range of data, including but not limited to years of tanning bed use, age at initial use, and types of tanning beds frequented, in order to estimate UVA/UVB ratios during exposure. The results of that study showed a 74% increase in melanoma risk among those who had ever tanned indoors versus those who had never done so. The researchers also revealed a strong dose–response relationship: Those who had tanned 10 or fewer times had a 34% higher risk of melanoma, and those who had tanned 100 times or more had a 272% higher risk, compared with those who had never tanned.^^15^^

What Lazovich’s team didn’t find, however, was evidence that melanoma risk increased with decreasing age of first tanning bed exposure. What drove the cancer risks higher, she explains, was exposure frequency. “Individuals who tan more often were at greatest risk regardless of the age they started to tan indoors,” Lazovich says. That’s important, she adds, because scientists still don’t know if younger people are biologically more sensitive to the carcinogenic effects of UV radiation, or if tanning during youth puts them at greater risk simply because they accumulate more exposures over time. It also is unclear what role burning plays in risk of melanoma; in Lazovich’s study, melanoma cases who used indoor tanning reported burning more frequently than controls who used indoor tanning.^^15^^

In a study that supplies some potential insight into some of those questions, Anne Cust, a senior lecturer at the Sydney School of Public Health in Australia, and colleagues looked at indoor tanning among 604 patients diagnosed with invasive cutaneous melanoma between the ages of 18 and 39. Compared with melanoma risk among those who did not use tanning beds, their findings showed that the risk of melanoma associated with 10 or more indoor tanning sessions was nearly 600% higher among patients diagnosed at or before the age of 29, compared with 60% higher among those diagnosed during ages 30–39.^^16^^

Those findings, Lazovich says, indicate that people diagnosed at younger ages might be genetically primed for melanoma “so that tanning triggers an illness that they might not otherwise have been diagnosed with until later in life.” But this idea, she says, needs more study.

Meanwhile, newer reports are adding to the evidence that indoor tanning boosts risks for non-melanoma skin cancers. Among them was a study in which Jiali Han, an associate professor in dermatology at Harvard University and Brigham and Women’s Hospital in Boston, and colleagues looked at cancer risk in relation to indoor tanning among 73,494 participants in the Nurses’ Health Study II. This long-term prospective study examined factors that affect women’s health, especially cancer risk. Han’s study revealed an 83% increased risk of BCC among women who used tanning beds most frequently during high school and college, and a 30% increased risk among those who used them most frequently between the ages of 25 and 35, compared with those who never used tanning beds. Significant associations were not observed for SCC or melanoma.^^17^^

A study published in December 2011 by Susan T. Mayne, a professor at the Yale School of Public Health, and colleagues backs up Han’s BCC findings. That study looked at 376 patients under age 40 who had been diagnosed with BCC—which Mayne says occurs rarely in this age group—and 390 controls. It revealed that, compared with those who did not use tanning beds, indoor tanning was associated with a 69% higher risk of BCC, with evidence of dose response for increasing sessions, years, burns, and hours of indoor tanning. Remarkably, BCC tumors showed up frequently on the trunk of the body, which is unusual given that these tumors more often occur on the face and neck in older people, Mayne says.^^18^^

Another study, this one by Portia T. Bradford and colleagues from the National Cancer Institute, detected rising rates of trunk melanoma on women under age 40. The authors cited changes in clothing patterns—namely bikinis and shirts that leave the back and front of the trunk exposed to the sun—as one potential factor in that trend but also pointed out that the use of indoor tanning beds is most common among young women.^^19^^

## Counterarguments

The Indoor Tanning Association has mounted two scientific arguments in defense of tanning beds. The first is that cancer risk from indoor—and outdoor—tanning derives more from burns than from UV exposure overall. “Indeed,” Overstreet says, “it is possible that moderate nonburning UV exposure actually reduces the risk of skin cancer through the mechanism of vitamin D.”^^20^^^^,^^^^21^^

Edward Giovannucci, a professor of nutrition at the Harvard School of Public Health, asserts that vitamin D appears to be important in various processes related to carcinogenesis. “For example, in animal models and in cell culture studies, it seems to be associated with reduced cell proliferation, more differentiation, and reduced angiogenesis,” he says. “Vitamin D may be important for these processes in some human cancers, though further studies to prove that these associations are causal are required.”

**Figure:**
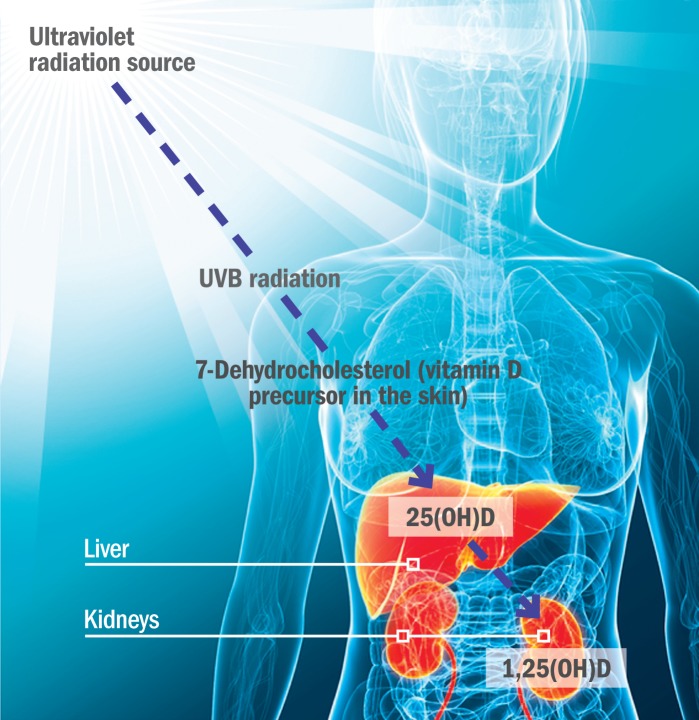
**UVB Exposure and Vitamin D** Cholesterol is a precursor to vitamin D. When UVB radiation hits the skin, it converts the compound 7-dehydrocholesterol to vitamin D3, which is carried to the liver and converted to 25-hydroxyvitamin D, or 25(OH)D. This form of vitamin D travels to the kidneys, where it becomes 1,25 dihydroxyvitamin D, or 1,25(OH)2D—the biologically active form of vitamin D. © 2012 Sebastian Kaulitzki/Shutterstock; ciarada/Shutterstock; Joseph Tart/EHP

Indoor tanning equipment used in the United States comes with labels mandated by the U.S. Food and Drug Administration (FDA) specifying the maximum amount of exposure permissible to avoid burns. Enforcement, however, is left to the states, says Overstreet; in states with lax oversight, he says customers may be “permitted to administer to themselves any amount of UV exposure they want, [and] they will often keep the UV exposure going until they ‘get some color’ in their skin, at which point they have already burned themselves.”

Overstreet says this is avoided in states with effective enforcement, where trained salon employees control the UV lamps, cutting them off when the proper exposure is obtained and enforcing 24- to 48-hour intervals between sessions. Overstreet adds that restricting access to indoor tanning salons could result in teenagers’ sunbathing or using home UV devices in search of a tan, potentially raising the likelihood of burns.

The association’s second argument is that the rise in melanoma rates might be artifactual and related more to changes in diagnostics and screening than to environmental factors such as indoor tanning. A key source behind that argument is Earl J. Glusac, a dermatopathologist at the Yale University School of Medicine. Glusac agrees that tanning beds pose a risk for skin cancers. But he’s skeptical that melanoma rates are actually rising significantly in the population. Glusac acknowledges that there could be an increased incidence of melanoma in small subsets of the population; however, he states that a true rising incidence of melanoma in the population as a whole would be accompanied by a corresponding rise in death rates from the disease, which is not the case.^^22^^

What has increased, Glusac says—citing greater awareness of melanoma and a public drive toward screening—is the biopsy rate for pigmented skin lesions, many of which will never spread and thus are unlikely to harm health. “There may be lesions that look like melanoma under the microscope that turn out to be biologically benign,” he posits. “We don’t know enough about the science yet to segregate these lesions, and they all get reported to the SEER database.”

## States Make the Call

Critics of indoor tanning point out that dietary supplements can address vitamin D deficiencies without the risks associated with UV exposure. Meanwhile, the tanning bed industry is being confronted with what appears to be a relentless sequence of studies that all lead to the same conclusion.

“There were some legitimate concerns with the first studies on skin cancer and indoor tanning,” says Jerod Stapleton, an assistant professor at the Robert Wood Johnson Medical School. “But the more recent studies in the literature are well designed, and together they comprise a body of evidence that’s hard to refute.”

Prospective research—in other words, a comparison of cancer incidence among people who tan indoors versus those who don’t in a study that goes forward in time—would go far in settling the debate, but such research is hampered by the fact that melanoma is so rare and by the complexity of potential confounding factors. And a controlled trial in which people are randomly assigned to tanning and nontanning exposure groups simply isn’t feasible for ethical reasons.

Scientists and the public alike are therefore left with retrospective evidence, and the question now devolves to how or whether the government should use that evidence to protect public health. For now, the majority of states are taking a precautionary approach. Only time will tell if it makes a difference in melanoma diagnoses.
